# Polycythemia vera revealed via a bladder tumor in a patient with erectile dysfunction: a case report

**DOI:** 10.1186/1752-1947-7-85

**Published:** 2013-03-27

**Authors:** Ahmed-Amine Bouchikhi, Mohammed Fadl Tazi, Soufiane Mellas, Driss Amiroune, Jalal Eddine Elammari, Abdelhak Khallouk, Mohammed Jamal El Fassi, Moulay Hassan Farih

**Affiliations:** 1 Urology Department, University Hospital of Fez, Fez, Morocco; 2 Rue Zag, Rce Andalous III, Quarier Al-Wafe, Fez, 30070, Morocco

**Keywords:** Bladder tumor, Bleeding, Erectile disorder, Hematuria, Polycythemia vera

## Abstract

**Introduction:**

Polycythemia vera is a polyglobular myeloproliferative syndrome related to the mutation of multipotent hemopoietic stem cells. This case report describes a patient whose bladder tumor was associated with polycythemia vera and erectile dysfunction. The association of bladder neoplasia with polycythemia vera and erectile dysfunction has not previously been reported in the literature.

**Case presentation:**

A 40-year-old Moroccan man was followed up for a bladder tumor which manifested with coagulant hematuria and a facial erythrosis with a hemoglobin level of 20.3g/L suggesting polycythemia vera. The patient also suffered from an erectile disorder. Considering the anesthesia difficulty due to polyglobulia, the patient was treated by bleeding. This treatment enabled the patient’s sexual performance to be improved and adjustment of his hemoglobin to a level allowing anesthesia, and hence surgical resection of his bladder tumor.

**Conclusion:**

Erectile dysfunction associated with polycythemia vera is elucidated by rheological disorders. Bleeding contributed to satisfactory sexual performance and facilitated treatment of polycythemia vera because it enabled anesthesia to be performed and hence the surgical resection of the bladder tumor.

## Introduction

Vaquez, in 1892, was the first to describe polycythemia vera [1]. Essential and acquired polyglobulia are myeloproliferative syndromes characterized by predominant clonal proliferation established in the erythroblastic lineage which is related to the mutation of multipotent hemopoietic stem cells [2].

We report the case of a patient whose bladder tumor was associated with polycythemia vera and erectile dysfunction. Considering the difficulty with anesthesia to polyglobulia, it was necessary to first treat the patient using repeated bleeding then the patient’s bladder tumor by surgery. It is notable that the patient’s erectile dysfunction stopped during bleeding treatment. Indeed, the association of a bladder tumor with polycythemia vera and erectile dysfunction has not previously been reported in the literature.

## Case presentation

A 40-year-old Moroccan man who was a chronic cigarette smoker was followed up for a bladder tumor which manifested 2 months earlier with coagulant hematuria. Resection of his bladder tumor was suggested. He reported headaches, dizziness, and a facial erythrosis. A general examination found a facial erythrosis without any peripheral adenopathy, hepatosplenomegaly, or physical masses. A clinical examination showed a middle prostate hypertrophy of 40g with lower vesicle flexibility.

The preoperative biological assessment showed a hemoglobin level of 20.3g/L, a hematocrit of 63.7%, red blood cells of 6,570,000/mm and a hyperleucocytosis of 14,000/mm.

An arterial blood gases study and imaging examinations including thorax radiography, spirometry, renal and hepatic ultrasound, thoracic and abdominal computed tomography (CT) scans, a CT scan of his brain and echocardiography showed no abnormalities. The cortisol rate of 8 hours, the urinary free cortisol, and the hemoglobin electrophoresis and markers’ dosage were without abnormalities. Hence, diagnoses of secondary polyglobulia and primary neoplasia were discarded. His total globular volume was found to be higher than the normal value at a rate of 40mL/kg confirming the essential characteristic of this polyglobulia; the result of a JAK2 mutation test was positive.

The patient was treated using iterative bleeding. It is notable that he neither drank alcohol nor presented with any particular stress and/or depressive characteristics. His blood pressure, glycemia and lipids assessment were all normal. The vesicoprostatic ultrasound and the testicular echo-Doppler were without abnormalities.

**Figure 1:**
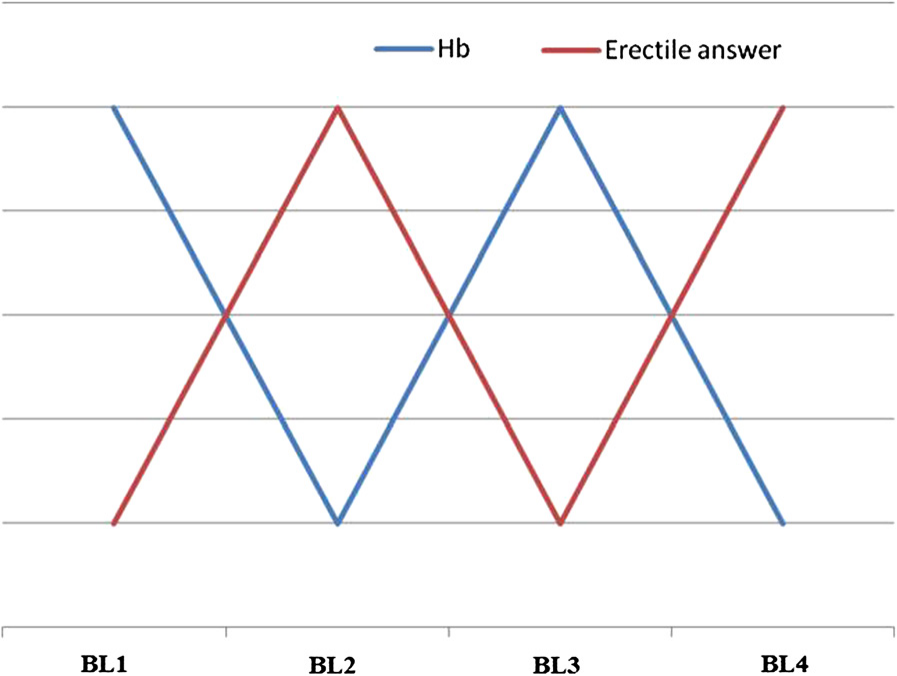
**Subjective fluctuations of the patient’s erectile responses compared to bleeding and correlated with hemoglobin levels.** The x**-**axis represents the bleeding while the y-axis represents the erectile response (red curve) with hemoglobin levels (blue curve). BL: Bleeding; Hb: Hemoglobin.

Indeed there was a correlation between the episodes of erectile disorder and the bleeding: significant improvement of sexual performance was noted and lasted 4 days after each bleeding session. Each time the bleeding therapy was stopped the erectile disorders restarted (Figure 1).

**Figure 2:**
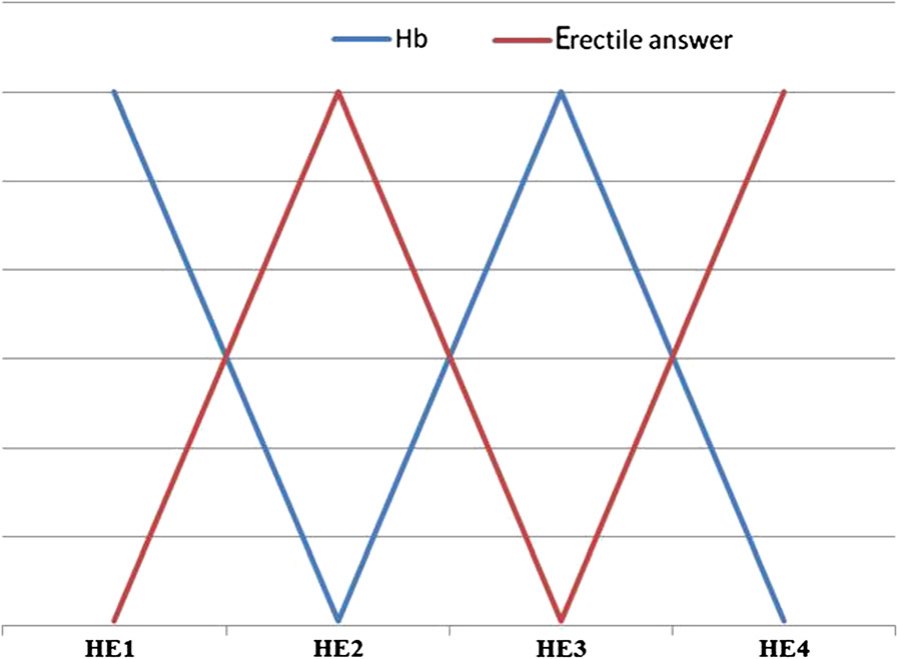
**Subjective fluctuations of the erectile response related to the hematuria episodes and correlated with hemoglobin levels.** The x**-**axis represents the hematuria episodes while the y-axis represents the erectile response (red curve) with hemoglobin levels (blue curve). HE = hematuria episode; Hb = hemoglobin.

The patient reported satisfactory sexual activity while presenting with hematuria (Figure 2). Since his marriage of one year earlier, the patient has been significantly affected by these disorders which required treatment with 5**-**phosphodiesterase inhibitors that unfortunately demonstrated very limited benefits.

## Discussion

The association of a bladder tumor with polycythemia vera and sexual dysfunction has not previously been described in the literature. In consideration of our patient’s erectile dysfunction history, he was a candidate for transurethral resection of bladder tumor. The preoperative assessment allowed demonstration of the polycythemia vera diagnosis.

The difficulty with anesthesia due to polyglobulia did not allow immediate and direct surgical treatment. Hence, the patient was treated using bleeding. Indeed the patient had satisfactory sexual activity during bleeding and hematuria. Therefore, we discuss the possible mechanisms of the disorder. The occurrence of the erectile dysfunction during essential polyglobulia is explained by microthromboses; vascular thromboses are known to arise during polycythemia vera, and involve all tissue territories and could be arterial or venous [2]. Although mostly partial, the cavernous body thromboses might contribute to complicate the polycythemia vera [3-5].

The microthromboses of polycythemia vera are worsened by slow circulation, blood hyperviscosity, thrombocytosis, hypercalcemia, and intima lesions caused by turbulent circulation and hyperviscosity [6]. Besides, it is known that the ultimate stage of vasculogenic erectile dysfunction results from the occlusion of cavernous arteries. The issued polyglobulia and the hypercoagulable blood initiate anoxia which mostly affects microcirculation with a possible involvement of microthromboses and relative tissue ischemia. The bleeding quickly reduces the viscosity. Hence, it allows the main cause of the functional signs and the vascular complications of this disease [2] to be suppressed. Indeed, there is no clear evidence that demonstrates a direct relationship between polycythemia vera and erectile dysfunction. However, the bleeding was demonstrated to simultaneously improve the polycythemia vera and the erectile dysfunction disorders. Larger scale studies involving physiopathological aspects could clarify the direct relation that might exist between erectile dysfunction and polycythemia vera because polycythemia vera is also a vascular disease.

These arguments could explain the correlation between erectile responses and bleeding output. They constitute a good explanation of the successful sexual activity of our patient during hematuria and bleeding. It should be noted that bleeding contributed to the decrease in his hemoglobin level, which enabled anesthesia to be performed to achieve the full surgical resection of his bladder tumor. Finally, larger studies might contribute to both the clarification and demonstration of the mechanisms involved in the pathology and hence lead to better and more efficient treatment approaches.

## Conclusion

Erectile dysfunction associated with polycythemia vera is elucidated by rheological disorders. Erectile disorders could originate from rheological disorders. Bleeding and hematuria allowed the patient's sexual performance to be improved and allowed opportunities for anesthesia. Hence, polycythemia vera therapy was facilitated and surgical resection of the patient’s bladder tumor became possible. Larger studies may better elucidate further mechanisms of this pathology and hence lead to better treatment approaches.

## Consent

Written informed consent was obtained from the patient for publication of this case report and any accompanying images. A copy of the written consent is available for review by the Editor-in-Chief of this journal.

## Competing interests

The authors declare that they have no competing interests.

## Authors’ contributions

AAB was the principal author and major contributor in writing the manuscript. MFT, SM, JEE, AK and DA analyzed and interpreted the patient data and reviewed the literature. MJE and MHF read and corrected the manuscript. All authors read and approved the final manuscript.
